# Chronic Consumption of Cranberries (*Vaccinium macrocarpon*) for 12 Weeks Improves Episodic Memory and Regional Brain Perfusion in Healthy Older Adults: A Randomised, Placebo-Controlled, Parallel-Groups Feasibility Study

**DOI:** 10.3389/fnut.2022.849902

**Published:** 2022-05-19

**Authors:** Emma Flanagan, Donnie Cameron, Rashed Sobhan, Chloe Wong, Matthew G. Pontifex, Nicole Tosi, Pedro Mena, Daniele Del Rio, Saber Sami, Arjan Narbad, Michael Müller, Michael Hornberger, David Vauzour

**Affiliations:** ^1^Norwich Medical School, Faculty of Medicine and Health Sciences, Norwich, United Kingdom; ^2^Department of Radiology, C.J. Gorter Center for High Field MRI, Leiden University Medical Center, Leiden, Netherlands; ^3^Human Nutrition Unit, Department of Food and Drug, University of Parma, Parma, Italy; ^4^Quadram Institute Bioscience, Norwich Research Park, Norwich, United Kingdom

**Keywords:** brain, flavonoids, cognition, BDNF, cerebral blood flow (CBF), arterial spin labelling (ASL), MRI, LDL-cholesterol

## Abstract

**Background:**

Ageing is highly associated with cognitive decline and modifiable risk factors such as diet are believed to protect against this process. Specific dietary components and in particular, (poly)phenol-rich fruits such as berries have been increasingly recognised for their protection against age-related neurodegeneration. However, the impact of cranberries on cognitive function and neural functioning in older adults remains unclear.

**Design:**

A 12-week parallel randomised placebo-controlled trial of freeze-dried cranberry powder was conducted in 60 older adults aged between 50 and 80 years. Cognitive assessment, including memory and executive function, neuroimaging and blood sample collection were conducted before and after the intervention to assess the impact of daily cranberry consumption on cognition, brain function and biomarkers of neuronal signalling.

**Results:**

Cranberry supplementation for 12 weeks was associated with improvements in visual episodic memory in aged participants when compared to placebo. Mechanisms of action may include increased regional perfusion in the right entorhinal cortex, the accumbens area and the caudate in the cranberry group. Significant decrease in low-density lipoprotein (LDL) cholesterol during the course of the intervention was also observed. No significant differences were, however, detected for BDNF levels between groups.

**Conclusions:**

The results of this study indicate that daily cranberry supplementation (equivalent to 1 small cup of cranberries) over a 12-week period improves episodic memory performance and neural functioning, providing a basis for future investigations to determine efficacy in the context of neurological disease. This trial was registered at clinicaltrials.gov as NCT03679533 and at ISRCTN as ISRCTN76069316.

## Introduction

The WHO estimates that by 2050, 22% of the world’s population will be aged over 60 years (1). This is a product of increasing life expectancy, which should be viewed as an exceptional achievement; yet the improvement remains somewhat overshadowed by the absence of preserved quality-of-life. With age, the predominant risk factor for numerous chronic and degenerative neurological conditions, maintaining quality-of-life represents a significant global challenge. Indeed, dementia incidence is projected to double every 20 years, affecting an estimated 152 million individuals by 2050 (2), placing considerable pressure upon already strained dementia care and disease management strategies. It is therefore imperative that proven effective solutions are developed to curb and manage current projections. The pathophysiological processes leading to the development of cognitive decline and dementias are complex and multifactorial, offering reasonable explanation for the failure of many pharmacological interventions to date (3). Future strategies may therefore benefit from a multifaceted approach. In addition to complex genetic predisposition, dementia risk is further influenced by numerous environmental factors such as diet, exercise and smoking status. Appealingly, these contributing environmental factors are often modifiable, and represent targets to address multiple underlying features of the disease process (4, 5). Therefore, achievable lifestyle interventions may represent an alternative approach to mitigate disease risk.

Epidemiological studies have reported that higher dietary intake of flavonoids is associated with slower rates of cognitive decline (6–8) and dementia (9). Foods rich in anthocyanins (responsible for imparting the red, purple and blue colour to several fruits and vegetables) and proanthocyanidins such as berries are consistently shown to improve cognition (10–13), and are further supported by a wealth of preclinical data (14–19), as well as emerging clinical evidence with fruit juices (20, 21) and freeze-dried fruit powder (22–24). Potential mechanisms include an enhancement of neuronal signalling and synaptic plasticity (17, 25), a modulation of glucose metabolism/insulin resistance (26, 27), a change in microbiota diversity and metabolism (28–30) along with regional increases in cerebral perfusion (31–34), however, further elucidation is required.

Cranberries (*Vaccinium macrocarpon*) are particularly rich in (poly)phenols such as anthocyanins, proanthocyanidins (both A- and B-type), flavonols and hydroxycinnamic acids (35), and are recognised for their antioxidant and anti-inflammatory effects (36–38) along with their capacity to modulate cardiometabolic endpoints (38–40). However, limited information is currently available regarding the evaluation of cranberries on cognitive performance, with the only previous study exploring the impact of cranberry intake on cognitive performance reporting no cognitive benefit of a cranberry juice consumption for 6 weeks in older adults with normal cognitive functions (41).

Here we report upon a single-centre, 12-week, double-blind, placebo-controlled parallel intervention study in which the impact of a freeze-dried cranberry powder intervention (equivalent of one small bowl) was examined in the context of cognitive health in healthy older adults (50–80 years). Cognitive health was determined using a battery of cognitive tests in combination with comprehensive biochemical and magnetic resonance imaging (MRI) assessments.

## Materials and Methods

### Study Participants

Healthy male and female older adults were recruited in this study through online recruitment databases (Join Dementia Research)^1^; existing research databases within the Norwich Medical School, University of East Anglia, where participants had previously consented to be contacted about research studies; and community-based advertising (e.g., recruitment posters, leaflets, talks). Participants were first pre-screened over the telephone for eligibility for the study using a screening questionnaire and were invited if they were aged between 50 and 80 years and presented with no subjective memory complaints as assessed by the Cognitive Change Index (CCI) questionnaire (42). Married couples who lived together were particularly targetted to reduce the variability in background diet patterns; however, participants were permitted to take part in the study on their own.

Individuals were excluded if they had any of the following condition: a diagnosis of any form of dementia or significant neurological condition, significant memory complaints, uncontrolled blood pressure, currently smoking or ceased smoking less than 6 months prior to enrolment, clinically diagnosed with psychiatric disorder, or currently on antidepressant or antipsychotic medication, diagnosed with a gastrointestinal disorder, or currently on any medication that alters the function of the gastrointestinal tract, chronic fatigue syndrome, liver disease, diabetes mellitus, or gall bladder abnormalities including gall bladder removal, history or MRI evidence of brain damage, including significant trauma, stroke, learning difficulties or developmental disorders, or a previous loss of consciousness for more than 24 h. In addition, participants were not eligible for the study if they were prescribed anticoagulant medicine such as warfarin, due to potential interactions with the active cranberry powder. Other exclusion criteria were restrictive or unbalanced diet and excessive alcohol consumption (>15 units/week). Participants were also excluded if they were identified as having a high flavonoid intake defined as >15 portions of flavonoid rich foods (fruit, vegetables, tea and coffee, fruit juice, dark chocolate, and cocoa) per day during the telephone screening (43).

For MRI measures, participants were not eligible to undergo the neuroimaging component of the study if they had a cardiac pacemaker, any metal surgical implants that would not be safe within the MRI machine, or experienced claustrophobia in small spaces. If participants were unable to undergo the neuroimaging component of the study, they were still able to take part in the other components of the study.

### Study Design

A single-centre, 12-week randomised, double-blind placebo-controlled parallel study design protocol was performed. Participants attended three visits in total: a screening visit (V0), a pre-intervention baseline visit (V1), and a follow-up visit at the end of the intervention (V2).

The screening visit (V0) involved obtaining informed consent, followed by collecting a fasted (>10 h) morning blood and urine sample, physical measurements (height, weight, and blood pressure). Basal blood pressure and heart rate was first collected after participants had been lying supine for 5 min, and then collected again upon standing. Participants were then provided with a standardised breakfast and underwent global cognitive screening using the Addenbrooke’s Cognitive Examination III (ACE-III). Participants were excluded if they scored <88 on the ACE-III, or had abnormal blood biochemistry, blood pressure or urine results indicative of a potential exclusion condition (e.g., diabetes, uncontrolled hypertension).

If participants passed the screening visit, they were invited to a pre-intervention baseline visit (V1). Following a standardised breakfast and other measures, as described above, participants completed a longer cognitive battery (2.5 h) including measures of processing speed, working memory, episodic memory and spatial navigation, and other experimental and perception tests to be published elsewhere, with a half hour break partway through testing to avoid fatigue. A 30-min MRI scan was also conducted either during this visit or within the same week of this visit. At the end of the baseline V1 visit, participants were provided with sachets of study powder, assigned to them using a computer-generated algorithm. The intervention was provided in the form of sachets (4.5 g each) of freeze-dried cranberry powder (Cranberry Institute, United States) designed to be incorporated into food and beverages (see Supplementary Table 1 for the product specifications for the freeze-dried cranberry powder). Participants were instructed to take two sachets per day, one in the morning and one in the evening, to maximise the physiological impact based on current understanding of bioavailability (44). The daily dosage of cranberry powder was roughly equivalent to consuming one cup or 100 g of fresh cranberries. This dosage was calculated to provide 281 mg proanthocyanidins, with increase of 20 mg flavonols and 59 mg anthocyanins per day (see Supplementary Methods and Supplementary Table 2). The placebo powder was designed to match the active cranberry powder for taste, colour, fructose, total sugar and calories and contained a blend of water, maltodextrin (CPC Maltrin M-180), citric acid, artificial cranberry flavour (Lorann oils), fructose, red colour (Lorann oils) and grape shade (Esco Foods) that had been freeze-dried. Participants were asked to return all remaining cranberry sachets at the end of the 12-week treatment period, with the number of leftover sachets being taken as one measure of compliance. Adherence to treatment was also determined by measuring total plasma (poly)phenol metabolites concentration as described previously thereafter.

Apart from the addition of the study powder, participants were asked not to modify their dietary intake in any further way, including any changes to their caloric intake. However, participants were asked to refrain from consuming any other non-essential supplements that could have a significant impact on the outcome measures for the duration of the study. Participants were also asked to fill in a validated, semi-quantitative SCG FFQ (version 6.6) (45) to account for their background diet.

The follow-up visit V2 was scheduled exactly 12 weeks following the baseline visit at the end of the intervention and was identical in procedures to the baseline visit. Fasted blood and urine samples were collected in addition to physical measurements, followed by the cognitive battery and MRI.

### Cognitive Assessment

Participants completed all of the following cognitive tests at the baseline and follow-up visits. The only exception to this was the ACE-III, which was conducted at the screening visit in the first instance to assess eligibility for the study (i.e., total score >88) and then repeated at the follow-up visit.

Global cognition was assessed using the ACE-III questionnaire, which covers domains including attention and orientation, memory, fluency, language and visuospatial functions (46). Executive functions and working memory were measured by using the Trail Making Test (TMT) (47), a short test of processing speed, attention, and set-shifting, and the Digit Span (DS) test, a subtest from the Weschler Adult Intelligence Scale–third edition (WAIS III) that assesses attention and short-term memory. DS is composed of two tasks administered independently of each other: “digits forward” and digits backward. For each “digits forward” item, participants are presented with a series of digits in increasing length and must immediately repeat them to the examiner in the same order as presented. For digits backward, the participant is required to repeat the number sequence in the reverse order. A composite executive function score was also calculated out of the ACE-III Category Fluency score (/7), the DS Backwards Raw Score (/14), and the Scaled Trails B from the TMT based on previously published normative data (48).

Memory was evaluated by using the Rey Complex Figure Test (RCF), a short measure of visual memory and visuospatial constructional ability (49). This study included the copy and 3-min recall trials of the test. A measure of verbal episodic memory was also measured using the delayed recall of the name and address on the ACE-III (score out of 7).

The Supermarket Test is a computer- and tablet-based assessment of spatial orientation that uses an ecological shopping environment (50). It includes a path integration test and measures (1) egocentric orientation, (2) short-term spatial memory, (3) heading direction, and (4) central (vs. boundary) based navigation preferences.

All tests were conducted using pen and paper, with the exception of the Supermarket Test which was administered using an Apple iPad.

### Magnetic Resonance Imaging

#### Data Acquisition

Magnetic resonance imaging scans were conducted in all eligible and willing participants at baseline and end of intervention and took approximately 30 min. In order to monitor structural brain information across the study, a T_1_-weighted 3D gradient-echo MR sequence was conducted at each testing visit. A T_2_-weighted fluid attenuated inversion recovery (FLAIR) scan was also conducted during the study visits. Arterial spin labelling (ASL) has previously been used to monitor changes in cerebral blood flow (CBF) in Alzheimer’s disease and mild cognitive impairment patients (51, 52).

All data were acquired on a 3 Tesla Discovery 750 w wide bore MR system (GE Healthcare, Milwaukee, WI, United States) with a 12-channel phased-array head coil for signal reception. After localisers, T_1_-weighted structural data were acquired using a 3D inversion-recovery fast spoiled gradient recalled echo (IR-FSPGR) sequence with repetition time (TR) = 7.7 ms; echo time (TE) = 3.1 ms; inversion time = 400 ms; field-of-view = 256 mm × 256 mm; acquired matrix = 256 × 256; 200 sagittal sections of 1 mm thickness; flip angle = 11°; and ASSET acceleration factor = 2 in the phase-encoding direction. Furthermore, a 3D T_2_-weighted FLAIR (T_2_w FLAIR) sequence was prescribed as follows: TR = 4800 ms; TE = 129 ms; inversion time = 1462 ms; field-of-view = 256 mm × 256 mm; acquired matrix = 256 × 256; 182 sagittal sections of 1 mm thickness; flip angle = 90°; an ARC acceleration factor of 2 in the phase-encoding direction; and a “HyperSense” compressed sensing subsampling factor of 2. The ASL scan consisted of a 3D spiral pseudo-continuous ASL (pCASL) acquisition with the following parameters: TE = 10.7 ms, TR = 4854 ms, 8 spiral interleaves with 512 sample points, field-of-view = 240 mm × 240 mm × 128 mm with a reconstructed resolution of 1.9 mm × 1.9 mm × 4 mm; post-label delay = 1500 ms, number of excitations = 3. Before analyses, all participant scans were visually inspected for significant head movements and artefacts.

#### Image Analysis

Voxel-Based Morphometry (VBM) was used on whole-brain T_1_-weighted scans using the VBM package in FSL (FMRIB Software Library, Oxford, United Kingdom) to confirm that there were no grey matter structural differences between the cranberry and placebo groups (53).

White matter hyperintensities (WMH) were rated using Multi-image Analysis GUI (Mango version 4.1, Research Imaging Institute, UTHSCSA, San Antonio, TX, United States) by one rater (EF). A well-established rating scale developed by Fazekas et al. (54) was used to qualitatively rate WMH in periventricular (PWMH) and deep (DWMH) regions using FLAIR images. WMH in the periventricular areas was rated as 0 = absent, 1 = “caps” or pencil-thin lining, 2 = smooth “halo,” or 3 = irregular, whereas DWMH were rated as 0 = absent, 1 = punctate foci, 2 = beginning confluence of foci, or 3 = large confluent areas.

For regional perfusion (ASL), equilibrium magnetisation (M_0_) and perfusion-weighted images were calculated in-line on the scanner workstation. All further analyses were performed using a processing pipeline written in bash and Python (v3.6, Python Software Foundation),^2^ which was run on the ADA high-performance computing cluster at the University of East Anglia. The pipeline closely resembled that used for the Alzheimer’s Disease Neuroimaging Initiative (ADNI)^3^ ASL sub-study, substituting FastSurfer for brain segmentation instead of FreeSurfer (55). In brief, M_0_ and perfusion-weighted images were scaled and used to calculate CBF maps in physical units of arterial water density (mL/min/100 g). T_1_-weighted data were then segmented using FSL’s FAST algorithm and the derived grey matter probability maps were used to register the ASL perfusion-weighted images to T_1_ space—*via* FSL’s FLIRT algorithm. ROIs from the FastSurfer segmentation were then used to determine ROI-wise CBF statistics: minimum maximum, mean, median, and standard deviation.

To visualise voxel-wise differences between groups, we performed higher-level general linear model analysis using FSL’s “randomise” permutation-testing tool. To facilitate this, structural T1-weighted images for all participants were first non-linearly registered to the Montreal Neurological Institute standard brain using FNIRT and the resulting transformation was applied to the ASL data. Difference images were then generated for each individual through subtraction of the baseline ASL scan from the post-intervention scan. An unpaired *t*-test was then performed on these data using “randomise” with threshold-free cluster enhancement to compare changes in perfusion between the cranberry and placebo groups.

### Biological Samples and (poly)Phenol Metabolites Analyses

A fasted blood sample was taken at the screening assessment visit and sent to the accredited pathology laboratories at the Norfolk & Norwich University Hospital (NNUH) for determination of markers of general health. Further blood samples were collected at baseline and follow-up in EDTA, SST and heparin vacutainer tubes (Becton-Dickinson, United Kingdom) for assessment of circulating metabolites, *APOE* genotype and blood biochemistry. Samples were immediately processed for serum/plasma, aliquoted and stored at −80°C until analysis. BDNF levels in plasma were assessed by ELISA (R&D Systems, United Kingdom) following the manufacturers’ instructions.

Plasma extraction of polyphenol metabolites was performed using microelution solid phase extraction (μSPE) according to validated protocols, with some modifications (56, 57). Briefly, plasma samples (350 μl) were diluted (1:1) with phosphoric acid 4% to reduce phenolic-protein interactions. Each sample (600 μl) was loaded on a 96 well μSPE plate, washed with water (200 μl) and 0.2% acetic acid (200 μl) and finally eluted with methanol (60 μl). The 96 well collection plates were directly put in the UHPLC autosampler for immediate analysis. Plasma samples were analysed through UHPLC DIONEX Ultimate 3000 fitted with a TSQ Vantage Triple Quadrupole Mass Spectrometer (Thermo Fisher Scientific Inc., San Jose, CA, United States) equipped with a heated-electrospray ionisation source (H-ESI-II; Thermo Fisher Scientific Inc.). Separations were performed with a Kinetex EVO C18 (100 mm × 2.1 mm), 2.6 μm particle size (Phenomenex). For UHPLC, mobile phase A was water containing 0.01% formic acid and mobile phase B was acetonitrile containing 0.01% formic acid. The gradient started with 5% B, keeping isocratic conditions for 0.5 min, reaching 95% B at 7 min, followed by 1 min at 95% B and then 4 min at the start conditions to re-equilibrate the column. The flow rate was set at 0.4 ml/min, the injection volume was 5 μl, and the column was thermostated at 40°C. The MS worked in negative ionisation mode with capillary temperature at 270°C, while the source was at 300°C. The sheath gas flow was 60 units, while auxiliary gas pressure was set to 10 units. The source voltage was 3 kV. Ultra-high-purity argon gas was used for collision-induced dissociation (CID). Compounds were monitored in selective reaction monitoring (SRM) mode, and characteristic MS conditions (S-lens RF amplitude voltage and collision energy) were optimised for each compound. Chromatograms, mass spectral data and data processing were performed using Xcalibur software 2.1 (Thermo Fisher Scientific Inc.). Quantification was performed with calibration curves of standards, when available; when not available, metabolites were quantified with the most structurally similar compound. Due to failure in collecting follow-up plasma, 14 volunteers were not considered for the calculations of plasma metabolite content.

### Statistical Analyses

Data were analysed using the Statistical Package for the Social Sciences (SPSS; v28.0), applying standard statistical thresholds (*p* < 0.05) and were tested for normality using the Shapiro–Wilk test. Mann–Whitney U Independent-Samples tests were employed to detect differences between demographic, anthropometric and biochemical data at baseline. One-way ANCOVA’s were used to detect baseline differences in cognition between groups controlling for age, education and gender. The impact of treatment on the cognitive outcomes of interest was established using mixed linear model with time and treatment as independent variables and with age, education and gender entered as covariates. Whole-brain differences in grey matter intensities were analysed between cranberry and placebo groups at baseline and follow-up with age added as a covariate. Periventricular and deep WMH were compared between cranberry and placebo groups at baseline and follow-up using ANCOVAs with age added as a covariate. Mean regional perfusion derived from ASL scans were analysed using mixed linear modelling with age entered as a covariate to determine and group × time interactions. Pearson correlations between significant cognition and regional perfusion at follow-up were also conducted.

Plasma metabolites, anthropometric and biochemical measures were compared between groups at baseline using non-parametric Mann-Whitney *U*-test analysis. Mixed linear modelling was used to detect within group differences between baseline and follow-up, as well as group × time interactions. Correlations analyses between plasma (poly)phenol metabolites and RCF delayed score along with regional blood perfusion from MRI ASL in regions found to be impacted by the cranberry intervention were conducted using non-parametric Spearman rank order. Unless otherwise stated, all results are presented as means (SD).

## Results

### Study Participants

**FIGURE 1:**
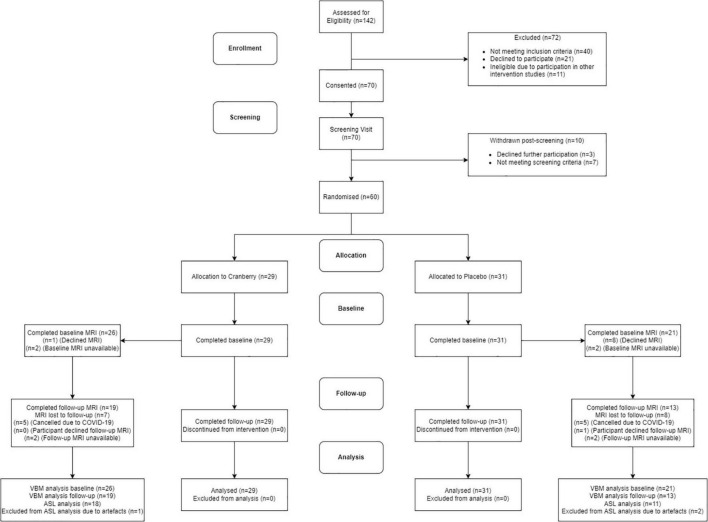
Consolidated standards of reporting trials (CONSORT) flowchart diagram. ASL, arterial spin labelling; MRI, magnetic resonance imaging; VBM, voxel-based morphometry.

Figure 1 shows a Consolidated Standards of Reporting Trials (CONSORT) flow diagram. Seventy participants were consented into the study. Of these participants, seven of them did not pass screening and three declined further participation between the screening and baseline visits. The final study population consisted of 60 participants who attended the baseline visit and commenced the intervention. All participants reported being in good health, not consuming any food supplements or medications that would interfere with the tested product. No participants discontinued the intervention or were withdrawn after they had attended the baseline visit, resulting in 60 participants completing the follow-up visit. There were no serious adverse events or protocol deviations reported during the study; however, there were two cases of participants experiencing dental changes, which were documented. Of the participants who were eligible for the intervention, 29 participants were randomised into the active cranberry treatment group and 31 into the control groups. All baseline values were within the physiological range and groups did not differ in age, education, distribution of gender or global cognitive performance at screening (Table 1).

**TABLE 1:** Demographic characteristics of the subjects at inclusion (*n* = 60).

Characteristics	Cranberry		Placebo		Sig. (p)
*N*	29		31		
Gender (M/F)	12/17		13/18		0.965

	**Mean**	**SD**	**Mean**	**SD**	

Age (years)	65.86	5.51	65.32	4.91	0.929
Education	14.38	2.60	14.61	3.01	0.610
ACE-III	96.58	2.39	96.10	2.80	0.644

*(Chi-Squared and Mann–Whitney U Independent-Samples Test).*

Overall, compliance was excellent based on returned empty sachets or unused study products and based on increased plasma total concentration of (poly)phenol metabolites. Specifically, plasma total concentration of (poly)phenol metabolites increased by 1.82 ± 0.57 μM in the cranberry group. No increase in plasma total (poly)phenol concentration was observed in the placebo group (Supplementary Figure 1).

Food-frequency data indicated there was no difference between the placebo and the cranberry groups on the macronutrient content of their background diets. However, a significant difference was observed (*p* = 0.02) in vitamin D concentration between the two groups, with the placebo group having a lower concentration (Supplementary Table 3). Although not reaching significance, the cranberry group presented with increased concentration of caffeine intake (*p* = 0.09). Flavonoid content was similar between the two groups with an average intake of 228 mg/day in the placebo group and 217 mg/day in the cranberry group (Supplementary Table 3).

### Cognitive Performance

There were no differences between cranberry and placebo groups at baseline on the ACE-III total score or on the sub-scores (Attention and Orientation, Memory, Fluency, Language, Address Delayed Recall and Category Fluency). No difference at baseline was also observed for RCF, Digits spans backward, Trail-making test A–B Scaled Score, or for the composite executive function score but there was a significant difference at baseline on visuospatial performance (*p* = 0.008, Table 2).

**TABLE 2:** Cognitive performance at baseline and follow-up, differences between groups at baseline (Mann–Whitney U Independent-Samples Test), and group × time interactions on linear mixed modelling.

			Baseline			Follow-up		Group × Time interaction
Measures		Treatment	M	SD	Sig. (p)	M	SD	Sig. (p)
ACE-III	Attention	Cranberry	17.65	0.72	0.219	17.66	0.55	0.467
		Placebo	17.35	1.11		17.60	0.62	
	Memory	Cranberry	24.97	1.18	0.726	25.10	1.05	0.498
		Placebo	24.84	1.70		25.67	1.95	
	Fluency	Cranberry	12.45	1.53	0.512	13.24	0.91	0.164
		Placebo	12.26	1.44		12.43	1.48	
	Language	Cranberry	25.79	0.49	0.305	25.76	0.51	0.401
		Placebo	25.58	0.54		25.83	0.46	
	Visuospatial	Cranberry	15.62	0.73	**0.008**	15.76	0.51	0.120
		Placebo	15.97	0.18		15.83	0.38	
	Address delayed recall	Cranberry	6.10	1.05	0.466	6.34	0.81	0.332
		Placebo	6.19	1.28		6.07	1.46	
	Category fluency	Cranberry	6.34	1.17	0.759	6.66	0.61	0.431
		Placebo	6.32	0.91		6.43	0.68	
RCF	Copy score	Cranberry	34.52	2.61	0.994	35.34	1.05	0.092
		Placebo	35.00	1.29		34.97	1.28	
	Delayed recall score	Cranberry	18.59	7.67	0.416	23.41	5.96	**0.028**
		Placebo	20.53	5.92		22.25	6.06	
Digit span	Forward raw score	Cranberry	11.28	2.28	0.437	11.41	2.01	0.309
		Placebo	10.84	2.28		11.39	2.50	
	Backward raw score	Cranberry	7.76	2.18	0.781	7.86	2.5	0.165
		Placebo	7.58	2.03		8.30	2.55	
TMT	A–B	Cranberry	37.41	21.84	0.416	35.93	16.22	0.639
		Placebo	33.29	16.89		34.14	13.73	
	B Scaled	Cranberry	14.83	1.79	0.756	15.03	1.66	0.127
		Placebo	15.13	1.73		15.37	1.97	
Executive composite score		Cranberry	28.93	3.50	0.964	29.34	3.73	0.430
		Placebo	29.03	3.41		29.93	3.45	

*ACE III, Addenbrooke’s cognitive examination III; RCF, Rey complex figure test; TMT, trail making test. Significant values p < 0.05 are in bold.*

At follow-up, a significant group × time interaction [*F*_(1, 55)_ = 5.060; *p* = 0.028] was observed in performance of the RCF test delayed recall such that the cranberry group showed a significant improvement in performance between baseline and follow-up compared to the placebo group. Linear mixed modelling to detect group × time interactions between the groups between baseline and follow-up did not reveal any differential impact of the intervention on groups pre- to post-treatment for the copy score nor the other cognitive tests (*p* > 0.05) (Table 2).

No significant differences were detected between groups on the egocentric, allocentric error or allocentric heading subtotals and totals of the Supermarket Test (Table 3). When the linear mixed modelling was run on these subtotals and totals, no significant group × time interactions were found (*p* > 0.05 in all cases).

**TABLE 3:** Spatial navigation performance at baseline and follow-up, differences between groups at baseline (Mann Whitney U independent-samples test), and group × time interactions on linear mixed modelling.

Measures			Baseline			Follow-up		Group × Time interaction
		Treatment	Mean	SD	Sig. (p)	Mean	SD	Sig. (p)
Supermarket test	Egocentric score section 1	Cranberry	3.32	1.84	0.383	3.76	1.96	0.906
		Placebo	3.67	1.76		3.92	1.91	
	Egocentric section 2	Cranberry	5.28	1.95	0.917	5.68	1.65	0.936
		Placebo	5.04	2.01		5.58	1.79	
	Egocentric total	Cranberry	8.60	3.29	0.656	9.44	3.29	0.885
		Placebo	8.71	3.36		9.50	3.27	
	Allocentric error section 1	Cranberry	11.72	4.35	0.324	12.54	8.31	0.267
		Placebo	13.00	4.94		11.96	5.81	
	Allocentric error section 2	Cranberry	14.28	3.00	0.164	15.47	10.54	0.481
		Placebo	14.11	4.55		13.27	4.76	
	Allocentric error total	Cranberry	13.00	3.14	0.964	13.98	8.88	0.325
		Placebo	13.55	4.47		12.70	4.70	
	Allocentric heading section 1	Cranberry	5.64	1.50	0.562	5.92	1.80	0.417
		Placebo	5.33	1.66		6.00	1.25	
	Allocentric heading section 2	Cranberry	5.60	1.50	0.489	5.84	1.34	0.697
		Placebo	5.38	1.41		5.79	1.29	
	Allocentric heading total	Cranberry	11.24	2.67	0.386	11.76	8.44	0.413
		Placebo	10.71	2.46		11.79	2.23	

### Magnetic Resonance Imaging

Among our study population, 47 participants were eligible and underwent the neuroimaging component of the study (26 in the cranberry group and 21 in the placebo group). Due to COVID-19 restrictions and reduced capacity of hospital facilities, 10 follow-up scans could not be conducted. An additional five follow-up scans could not be scheduled due to participants (*n* = 1) or scanning facilities (*n* = 4) being unavailable during the critical follow-up time window. Additionally, ASL data for 2 baseline scans and 1 follow-up scan were not usable due to severe motion artefacts.

There were no differences in whole brain grey matter intensity between cranberry and placebo groups found at either baseline or follow-up scans as assessed by the VBM package in FSL with a *p* threshold set at 0.05 with age added as a covariate. No statistically significant differences were observed between groups in periventricular WMH between the cranberry and placebo groups at baseline (*p* = 0.688) or follow-up (*p* = 0.833), or for deep WMH at baseline (*p* = 0.693) or follow-up (*p* = 0.723) in ANCOVA’s with age added as a covariate. Two participants were rated as “3” for periventricular WMH (i.e., “irregular”), with the remaining participants being rated between 0–2.

**FIGURE 2:**
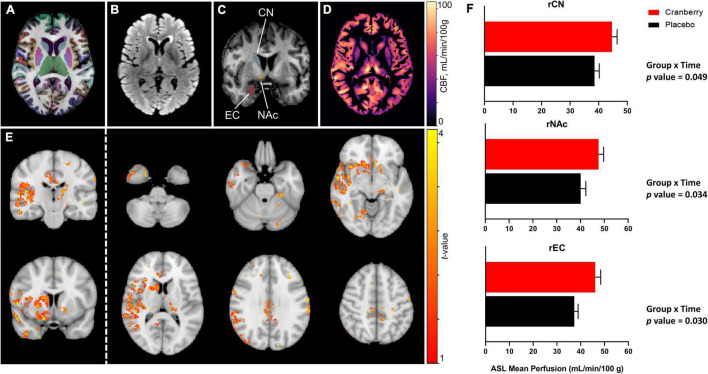
Representative magnetic resonance imaging data following 12-week consumption of a cranberry extract or a placebo. **(A)** Axial view of FastSurfer cortical and subcortical segmentations superimposed on a T1-weighted image; **(B)** axial T2-weighted fluid attenuated inversion recovery (FLAIR) image; **(C)** a coronal view of the T1-weighted image indicating regions that showed significantly increased perfusion after 12 weeks consumption of a cranberry extract—namely, from superior to inferior, the right caudate nucleus, accumbens area, and entorhinal cortex; **(D)** an axial cerebral blood flow (CBF) map, in the T1 space, derived from arterial spin labelling data; **(E)** *t*-value maps overlaid on a standard brain show trends to increased perfusion in the cranberry group as compared to the placebo group, in similar regions to those indicated in the ROI-wise analyses; and **(F)** differences in mean blood perfusion for the right caudate nucleus (rCN), right nucleus accumbens (rNAc), and right entorhinal cortex (rEC), with *p*-values represented for group × time interaction effects between cranberry and placebo groups from baseline to follow-up.

Figures 2A–D depict representative magnetic resonance imaging data. Mean regional CBF from ASL for the cranberry and placebo groups at baseline and follow-up are summarised in Supplementary Table 4. No significant differences in regional perfusion between cranberry and placebo groups were detected at baseline (*p* > 0.05). ASL group analysis with FSL’s “randomise” tool indicated voxels covering similar volumes to the ROI’s indicated in the ROI-wise analyses (Figure 2E). Mixed linear modelling controlling for age and education detected significant group × time interactions for the right caudate [*F*_(1, 29.275)_ = 4.207, *p* = 0.049], right accumbens area [*F*_(1, 31.744)_ = 4.916, *p* = 0.034], and right entorhinal cortex [*F*_(1, 30.558)_ = 5.202, *p* = 0.030]. All models involved an increase in perfusion between baseline and follow-up in the cranberry group compared to a relative decrease in perfusion over time in the placebo group (Figure 2F).

As a significant group × time interaction was found for the delayed recall of the RCF, a correlation analysis was performed between the follow-up RCF delay scores and follow-up regional perfusion data, however, no significant correlation was found for either group (*p* > 0.05).

### Biological Samples and Polyphenol Metabolites Analyses

There was a group × time effect on cholesterol, such that total, low-density lipoprotein (LDL) but not high-density lipoprotein (HDL) cholesterol decreased over the 12 weeks in cranberry but not in the placebo group [LDL; *F*_(1, 40)_ = 4.100; *p* = 0.048]. Although not reaching significance, a trend toward a decrease in total cholesterol was also observed in the cranberry group [*F*_(1, 42)_ = 4.073; *p* = 0.050] when compared to the placebo group (Table 4).

**TABLE 4:** Blood biochemistry, blood pressure, heart rate and anthropometry at baseline and follow-up, differences between groups at baseline (Mann-Whitney U Independent-Samples Test), and group × time interactions on linear mixed modelling.

			Baseline			Follow-up		Group × Time interaction
Measures		Treatment	Mean	SD	Sig. (p)	Mean	SD	Sig. (p)
Biochemistry	Cholesterol (mmol/L)	Cranberry	5.6	1.1	0.98	5.4	1.2	0.050
		Placebo	5.5	1.0		5.4	1.1	
	HDL cholesterol (mmol/L)	Cranberry	1.7	0.4	0.98	1.7	0.3	0.428
		Placebo	1.6	0.4		1.6	0.4	
	LDL cholesterol (mmol/L)	Cranberry	3.5	1.0	0.81	3.2	1.0	**0.048**
		Placebo	3.4	0.9		3.3	1.2	
	Triglyceride (mmol/L)	Cranberry	1.0	0.4	0.96	1.1	0.4	0.518
		Placebo	1.2	0.6		1.1	0.5	
	Glucose fasting (mmol/L)	Cranberry	4.7	0.4	0.98	4.6	0.4	0.963
		Placebo	4.8	0.5		4.7	0.5	
	ALT (U/L)	Cranberry	17.8	5.2	0.90	18.6	7.6	0.812
		Placebo	17.3	8.5		17.6	6.5	
	AST (U/L)	Cranberry	22.4	4.6	0.83	22.8	6.8	0.459
		Placebo	24.6	3.6		22.4	5.1	
	Alkaline phosphatase (U/L)	Cranberry	69.3	17.7	0.24	69.2	16.1	0.860
		Placebo	73.8	22.0		73.0	18.0	
	Creatinine (μmol/L)	Cranberry	72.9	12.4	0.96	73.4	10.5	0.609
		Placebo	73.1	14.0		71.3	11.6	
	Total bilirubin (μmol/L)	Cranberry	11.2	4.1	0.45	10.3	3.8	0.110
		Placebo	14.1	4.9		15.1	6.1	
	Total protein (g/L)	Cranberry	71.0	3.8	0.81	70.7	4.0	0.669
		Placebo	71.9	3.5		72.0	3.3	
	Albumin (g/L)	Cranberry	40.6	2.3	0.86	40.4	3.1	0.133
		Placebo	40.0	2.3		40.8	2.2	
	Globulin (g/L)	Cranberry	30.4	3.8	0.71	30.2	3.7	0.803
		Placebo	31.8	3.1		31.3	3.2	
	Urea (mmol/L)	Cranberry	5.1	1.0	0.96	5.0	1.1	0.862
		Placebo	4.9	1.0		4.7	1.2	
	Calcium (mmol/L)	Cranberry	2.3	0.1	0.98	2.3	0.1	0.198
		Placebo	2.4	0.1		2.4	0.1	
	Adjusted calcium (mmol/L)	Cranberry	2.4	0.1	>0.99	2.4	0.1	0.757
		Placebo	2.4	0.1		2.4	0.1	
	Phosphate (mmol/L)	Cranberry	1.0	0.2	>0.99	1.0	0.2	0.947
		Placebo	1.0	0.2		1.0	0.2	
	Bicarbonate (mmol/L)	Cranberry	27.0	3.2	0.77	26.7	2.2	0.341
		Placebo	25.9	2.2		26.1	1.9	
	Na (mmol/L)	Cranberry	139.5	2.3	0.19	139.7	2.4	0.496
		Placebo	134.5	2.9		138.0	2.2	
	K (mmol/L)	Cranberry	4.5	0.3	>0.99	4.4	0.2	0.996
		Placebo	4.5	0.3		4.5	0.4	
Blood pressure and heart rate	Diastolic (mm Hg)	Cranberry	79.3	9.9	0.60	81.2	10.7	0.786
		Placebo	81.3	11.2		82.9	10.5	
	Systolic (mm Hg)	Cranberry	139.0	17.4	0.85	134.9	18.5	0.521
		Placebo	139.7	18.3		132	13.6	
	Heart rate (bpm)	Cranberry	62.7	8.6	0.61	61.5	8.1	0.209
		Placebo	61.6	8.2		62.9	10.2	
Anthropometry	BMI (Kg/m^2^)	Cranberry	24.9	4.0	0.93	24.1	3.0	0.305
		Placebo	25.0	5.9		25.7	3.9	
	Weight (Kg)	Cranberry	71.5	15.2	0.88	68.5	11.8	0.271
		Placebo	72.1	18.5		73.7	13.8	

*ALT, alanine transaminase; AST, aspartate transaminase; BMI, body mass index; HDL, high-density lipoprotein; K, potassium; LDL, low-density lipoprotein; Na, sodium. Significant values p < 0.05 are in bold.*

Investigating our data further, we observed a strong interindividual variability with male participants possibly experiencing a greater benefit from the cranberry intake. Indeed, a steep but not significant decrease in BMI (from 27 ± 4.2 to 24.5 ± 2.5 kg/m^2^) in male participants was observed following 12 weeks of cranberry intake. This decrease also applied to fasting blood glucose and systolic blood pressure along with an increase in HDL cholesterol (Supplementary Figure 2).

There was no significant difference in BDNF concentration following cranberry intake for 12 weeks when compared to placebo (*p* = 0.7119) although concentrations were higher than in the placebo group at follow up (Supplementary Figure 3). High concentrations of BDNF in the cranberry group at baseline may be explained by higher concentration of caffeine intake in this group (Supplementary Table 2). BDNF at the follow-up visit did not correlate significantly with CBF in any treatment group (*p* > 0.05).

There were no significant differences in plasma total (poly)phenol metabolites at baseline, apart from the flavonol kaempferol-3-glucuronide which presented higher concentration in the placebo group [Mann Whitney-U (1, 43) = 351.00, *p* = 0.026; Table 5]. At follow-up, there were significant increases in 4-methylcatechol-sulphate, hippuric acid, caffeic acid and total (poly)phenol metabolites in the cranberry group when compared to the placebo (Table 5). No significant correlation between circulating (poly)phenol metabolites, RCF scores or regional blood perfusion at follow-up were observed (*p* > 0.05), indicating an indirect effect of the cranberry treatment on cognitive performance and brain perfusion.

**TABLE 5:** Plasma (poly)phenol metabolites at baseline and follow-up visits in μmol/L.

Metabolite	Group	Baseline (μ mol/L)		Baseline difference	Follow-up (μ mol/L)		Group × Time
		M	SD	*p*	M	SD	*p*
Kaempferol-3-glucuronide (Flavonols)	Cranberry	0.017	0.018	**0.026**	0.019	0.020	0.274
	Placebo	0.033	0.034		0.028	0.022	
4-Methylcatechol-sulphate (Catechols)	Cranberry	0.033	0.016	0.617	0.042	0.015	**0.003**
	Placebo	0.032	0.012		0.024	0.010	
4-Hydroxybenzaldehyde (Benzaldehydes)	Cranberry	0.013	0.006	0.609	0.019	0.013	0.307
	Placebo	0.013	0.006		0.018	0.009	
Hippuric acid	Cranberry	2.82	1.58	0.892	4.29	1.94	**0.002**
	Placebo	2.76	1.12		2.53	1.78	
4-Hydroxyhippuric acid	Cranberry	0.039	0.020	0.540	0.043	0.025	0.508
	Placebo	0.043	0.023		0.041	0.025	
4-Hydroxybenzoic acid	Cranberry	0.024	0.022	0.056	0.027	0.012	0.358
	Placebo	0.032	0.079		0.038	0.054	
Benzoic acid-4-sulphate	Cranberry	0.023	0.023	0.107	0.028	0.024	0.192
	Placebo	0.015	0.009		0.014	0.009	
Benzoic acid-3-sulphate	Cranberry	0.026	0.038	0.609	0.020	0.021	0.588
	Placebo	0.015	0.014		0.012	0.011	
Caffeic acid (3′,4′-Dihydroxycinnamic acid)	Cranberry	0.021	0.012	0.856	0.032	0.014	**0.005**
	Placebo	0.020	0.008		0.019	0.013	
Ferulic acid-4-glucuronide	Cranberry	0.018	0.020	0.927	0.025	0.015	0.061
	Placebo	0.016	0.015		0.012	0.010	
3-Hydroxyphenylacetic acid (Phenylacetic acids)	Cranberry	0.078	0.041	0.496	0.087	0.042	0.811
	Placebo	0.068	0.033		0.074	0.028	
3-(3′-Hydroxyphenyl)propanoic acid (Phenylpropanoic acids)	Cranberry	0.051	0.043	0.115	0.039	0.036	0.166
	Placebo	0.077	0.054		0.041	0.049	
5-(Phenyl)-γ-valerolactone-methoxy-glucuronide (3′,4′) (Phenyl-γ-valerolactones)	Cranberry	0.018	0.011	0.751	0.017	0.010	0.190
	Placebo	0.017	0.009		0.013	0.008	
Total metabolites	Cranberry	3.18	1.68	0.820	4.69	2.01	**0.002**
	Placebo	3.14	1.20		2.87	0.321	

*Significance of baseline group differences was determined by non-parametric Mann-Whitney U independent samples tests, and main effects of time within group between baseline and follow-up along with interactions between group and time was determined using linear mixed modelling. Significant values p < 0.05 are in bold.*

No participants were found to carry two copies of the *APOE*-4 mutation. Furthermore, there were no differences between the cranberry and placebo groups for distribution of *APOE* genetic types, χ2 (3) = 2.60, *p* = 0.457 (Table 6).

**TABLE 6:** *APOE* genetic status of participants in the cranberry and placebo groups, and overall totals.

*APOE* genotype	Cranberry	Placebo	Total
E2/E3	3	4	7 (11.67%)
E2/E4	0	2	2 (3.33%)
E3/E3	21	22	43 (71.67%)
E3/E4	5	3	8 (13.33%)

*APOE, Apolipoprotein E.*

## Discussion

This investigation reports the effect of long-term cranberry supplementation (12-week placebo-controlled intervention) upon cognitive performance and brain health. Daily supplementation with freeze-dried cranberry extract (equivalent to one cup of fresh cranberries) led to significant improvements in episodic memory performance, which coincided with increased perfusion of key neural areas which support cognition in older adults. Our results are in direct contrast to a previously conducted clinical investigation in which no significant change in memory performance was established following cranberry intake (41). The discordance between these results likely relates to experimental inconsistencies such as duration of the intake (6 vs. 12 weeks) or product formulation (cranberry juice vs. freeze-dried whole cranberry powder).

Surprisingly, the cranberry intervention had no further impact upon additional neurocognitive domains. Working memory and executive functioning (including the executive functioning composite score) remained unaltered despite the contrary being reported by others investigating flavonoid-rich juices e.g., blueberry, Concord grape juice and orange juice (21). This could in part relate to the distinct (poly)phenolic composition of each distinct intervention. Conversely, this may be a product of cognitive test choice, with tests such as the DS backward and trail making A and B believed to be less sensitive in detecting changes in executive functioning in non-cognitively impaired adults (58). In agreement with our results, a longer-term 12-week intervention with wild blueberry juice (20) led to similar improvements upon episodic memory performance in older adults. These two studies highlight that longer duration of supplementation is required to establish episodic memory enhancement associated with high anthocyanin and proanthocyanidin containing berries.

As anticipated, the cranberry intervention had no impact upon differences in structural grey matter between groups, nor did it influence differences in WMH over the 12-week period of investigation. However, in line with results suggesting that the cranberry intervention led to improved episodic memory performance, differences in perfusion in response to the intervention were detected between cranberry and placebo groups in key cerebral regions supporting memory consolidation and retrieval (59). A relative increase in perfusion was detected in the cranberry group between baseline and follow-up compared to the placebo group in medial temporal (entorhinal) and prefrontal (orbitofrontal) regions, as well as in the nucleus accumbens, which would provide optimal distribution of the essential nutrients for neuronal activity, such as oxygen and glucose (60).

The pathophysiological processes leading to neurodegeneration, like many other diseases, are proposed to involve the dysfunction of multiple systems within the body. Neurodegeneration is hypothesised to be characterised by progressive changes in several interlinked cellular and molecular mechanisms, including chronic neuroinflammation, oxidative stress and metabolic imbalances, as well as loss of vascular integrity and function, deposition of aggregated proteins, mechanisms that underlie not only pathological but also normal brain ageing, resulting in loss of neural plasticity and neuronal death (61). However, the exact nature and results of these processes are still being elucidated. Treatments that can target and slow down these processes would be valuable in counteracting brain ageing and cognitive decline. Results from intervention studies involving humans remain less prevalent, and as such findings that lend support to the causal effects of berry (poly)phenols on the prevention of age-related cognitive decline and dementia from interventional studies remain sparse, and further attention needs to be dedicated to determining the causality of flavonoid consumption on improving cognition and preventing dementia.

Although it was anticipated that BDNF may increase in the cranberry group (in line with improved cognition) and as reported for other flavonoids (62), such results were not significant. In particular, a high concentration of BDNF was measured at baseline which masked the overall impact of cranberries at follow-up. Such increased concentration may be related to participants’ higher consumption of caffeine in this group at baseline. During our analysis, we also observed a significant decrease in LDL cholesterol following cranberry intake, along with a trend in total cholesterol decreases. Such results are in agreement with a previous study demonstrating that cranberry supplements were effective in reducing atherosclerotic cholesterol profiles, including LDL cholesterol and total cholesterol levels (63). Interestingly, in our study, cranberry intake seemed to better benefit older male participants where a steep decrease in body weight and associated parameters [fasting glucose, high-density lipoprotein (HDL) cholesterol, blood pressure] were better controlled. Such results are in agreement with data demonstrating the anti-obesogenic impact of cranberries, although most of the information is derived from animal studies (64). Further studies would be necessary to confirm such findings in well controlled clinical studies.

In addition to biochemical measures, the circulating plasma (poly)phenol metabolites indicated that both groups were well matched at baseline, apart from flavanols which were higher in the placebo group before the intervention. The results of the background diet questionnaires also indicated that the placebo group had a higher average intake of flavonols, although this difference between groups was not significant. The high molecular weight fraction in our study represented 70.4%, so high concentrations of phenolic metabolites/catabolites were not expected after overnight fasting. There were, however, increases in total metabolites in the cranberry group as a result of the intervention, which appeared to be driven largely by significant increases in catechols and hippuric acid. Further and contrary to expectations, plasma (poly)phenol metabolite concentrations did not relate to either RCF delay score or regional blood perfusion within the regions found to be differentially changed between groups as a result of the intervention. It is therefore possible that the mechanisms underpinning the changes in cognition and regional blood perfusion in the brain were not the direct interaction between these metabolites and neural targets.

The relatively small sample size of this intervention may have been a limiting factor particularly with regards to having sufficient power to detect significant differences in both cognition and brain perfusion. Indeed, regarding the neuroimaging data, several regions showed a trend for perfusion differences such as the insula and the medial orbitofrontal cortex, which may have reached significance if more participant scans were available. Only a subsection of the study sample was able to have complete baseline and follow-up MRI scans due to practical constraints and the impact of COVID-19 lockdowns on hospital imaging facilities during the critical follow-up window for several (*n* = 14) patients. Furthermore, although it was not reported by any participants, other health conditions which may have influenced cognitive results such as sleep apnoea were not systematically excluded in this study. It is also important to note that the cranberries also contain other health-promoting compounds and nutrients, including fibre and other nutrients, making it sometimes difficult to determine whether it is in fact these specific polyphenols producing the health effects. For example, nutrients such as fermentable fibres can influence gut microbial metabolism of polyphenols (65). Furthermore, other mechanisms such as chronic neuroinflammation, mitochondrial function and compromised vascular integrity and function are increasingly becoming understood to be key mechanisms which also contribute to age-related cognitive decline and neurodegenerative conditions and provide targets for interventions to curtail the disease processes contributing to age-related neurodegeneration [for review, see Flanagan et al. (66)]. Indeed, these mechanisms are also among the targets of nutritional interventions including those involving high-polyphenol foods, particularly in light of their suggested bidirectional relationships with the function of gut microbiota. Similarly, markers of other factors that could be impacted by cranberry intake and could also relate to neurodegeneration such as chronic infection (67) were not measured. The focus of the intervention discussed in this study was the impact of a long-term cranberry intervention on cognition and brain function, and as such the investigation of the impact on these additional mechanisms, although important, fell outside the scope of this study. Finally, as the physicochemical properties and dietary intake forms could impact the absorption and bioactivity of nutrients (68) contained in the cranberry powder, and as such could be controlled in future investigations to ensure that the effectiveness of the cranberry is not impacted by different methods of incorporating it into the diet.

These findings are, however, certainly encouraging that sustained intake of cranberry over a 12-week period produced significant improvements in memory and neural function in older adults who were cognitively healthy. Future studies investigating whether these changes translate to a clinical population of cognitively impaired adults in the context of neurodegenerative conditions such as mild cognitive impairment or dementia is warranted based on these results. Determining whether these changes are sustained following the cessation of intake, for how long and to what degree would also be of interest. Replication of this study in a larger sample size might also produce more robust results.

## Data Availability Statement

The raw data supporting the conclusions of this article will be made available by the authors, without undue reservation.

## Ethics Statement

The studies involving human participants were reviewed and approved by the University of East Anglia’s Faculty of Medicine and Health Sciences Ethical Review Committee (Reference: 201819–039) and the Health Research Authority (IRAS number: 237251). The patients/participants provided their written informed consent to participate in this study.

## Author Contributions

AN, MM, MH, and DV contributed to the conception and design of the study, funding acquisition, and wrote the manuscript. EF contributed to the day-to-day management of the study, data and sample acquisition, formal analysis and wrote the manuscript, and had primary responsibility for final content. MP analysed BDNF concentrations in biological samples. DC, CW, RS, and SS contributed to the MRI data analysis and statistical analyses. NT, PM, and DD analysed the (poly)phenol content in biological samples and extracts. All authors contributed to the manuscript revision, read, and approved the submitted version.

## Conflict of Interest

DV, MH, MM, and AN received funding from the Cranberry Institute. The remaining authors declare that the research was conducted in the absence of any commercial or financial relationships that could be construed as a potential conflict of interest.

## Publisher’s Note

All claims expressed in this article are solely those of the authors and do not necessarily represent those of their affiliated organizations, or those of the publisher, the editors and the reviewers. Any product that may be evaluated in this article, or claim that may be made by its manufacturer, is not guaranteed or endorsed by the publisher.

## Footnotes

1
https://www.joindementiaresearch.nihr.ac.uk/

2
www.python.org

3
adni.loni.usc.edu
