# Supplementary material for: Chronic Consumption of Cranberries (Vaccinium macrocarpon) for 12 Weeks Improves Episodic Memory and Regional Brain Perfusion in Healthy Older Adults: A Randomised, Placebo-Controlled, Parallel-Groups Feasibility Study
Source: Front Nutr. 2022 May 19;9:849902. doi: 10.3389/fnut.2022.849902 (PMC9160193; doi:10.3389/fnut.2022.849902)
Supplement: Supplementary file 1 [file Data_Sheet_1.docx]

***Methods***

***Identification and quantification of phenolic compounds in the cranberry powder by uHPLC–MS^n^ analysis***

Two packages of cranberry powder were extracted in triplicate as reported previously (Mena et al., 2016), with some modifications. Briefly, 50 mg of powder were added with 1 mL of 50% aqueous methanol acidified with formic acid (0.1%). The solution was vortexed for 1 minute, sonicated for 25 min, vortexed for 1 minute, and centrifuged at 12,000 rpm for 10 min at 4°C. The supernatant was collected. The pellet was re-extracted twice using 0.5 mL of the same solvent, following the same procedure, and the three supernatants were pooled. Finally, extracts of the powders were diluted with acidified water (0.1 formic acid) (1:20, 1:10, 1:5 and 1:2) before uHPLC-MSn analysis.

The identification and quantification of the (poly)phenols present in cranberry powder was performed using an untargeted, full-scan, MS2 analysis. Powder extracts were analysed by ultra-high performance liquid chromatography (uHPLC) coupled with mass spectrometry (MS), using an Accela uHPLC 1250 apparatus equipped with a linear ion trap MS (LIT-MS) (LTQ XL, Thermo Fisher Scientific Inc., San José, CA, USA), fitted with a heated-ESI (H-ESI-II) probe (Thermo Fisher Scientific Inc.). Separation was carried out by means of Kinetex EVO C18 column (100 x 2.1 mm; 2.6 µm particle size; Phenomenex, CA, USA) installed with a precolumn cartridge (Phenomenex). Phenolic compounds were analysed in negative ionization mode, with the exception of anthocyanins, which were detected in positive ionization mode. For both methods, mobile phase, pumped at a flow-rate of 0.4 mL/min, consisted of a mixture of acidified acetonitrile (0.1% formic acid) (solvent A) and 0.1% aqueous formic acid (solvent B). Following 0.5 min of 5% solvent A in B, the proportion of A was increased linearly to 51% over a period of 8.5 min. Solvent A was increased to 80% in 0.5 min, maintained for 2 min and then the start conditions were re-established in 0.5 min and maintained for 5 min to re-equilibrate the column (total run: 17 min). For negative mode, the H-ESI-II interface was set to a capillary temperature of 275 °C and the source heater temperature was 200 °C. The sheath gas (N2) flow rate was set at 40 (arbitrary units) and the auxiliary gas (N2) flow rate at 5. The source voltage was 4 kV, the capillary voltage was -42 V and tube lens voltage was -118 V. For anthocyanin analysis, the H-ESI-II interface was set to a capillary temperature of 275 °C and the source heater temperature was 300 °C. The sheath gas (N2) flow rate was set at 40 (arbitrary units) and the auxiliary gas (N2) flow rate at 5. The source voltage was 4.5 kV, and the capillary voltage and tube lens voltage were +20 and +95 V, respectively. Both in positive and in negative ionization mode, a collision induced dissociation (CID) equal to 35 (arbitrary units) was used to obtain MS2 fragmentation. Quantification was performed using calibration curves built with pure standards, when available, or using the curves built with the most structurally similar compound.

**Supplementary Figure S1:** Plasma concentration of total flavonoids at baseline and following 12 weeks consumption of either a cranberry extract or a placebo. ***p<0.001.

**Supplementary Figure S2:** Impact of 12 weeks consumption of cranberry powder on BMI, fasting glucose HDL cholesterol and systolic blood pressure in both male and female participants. No significant changes were observed.

**
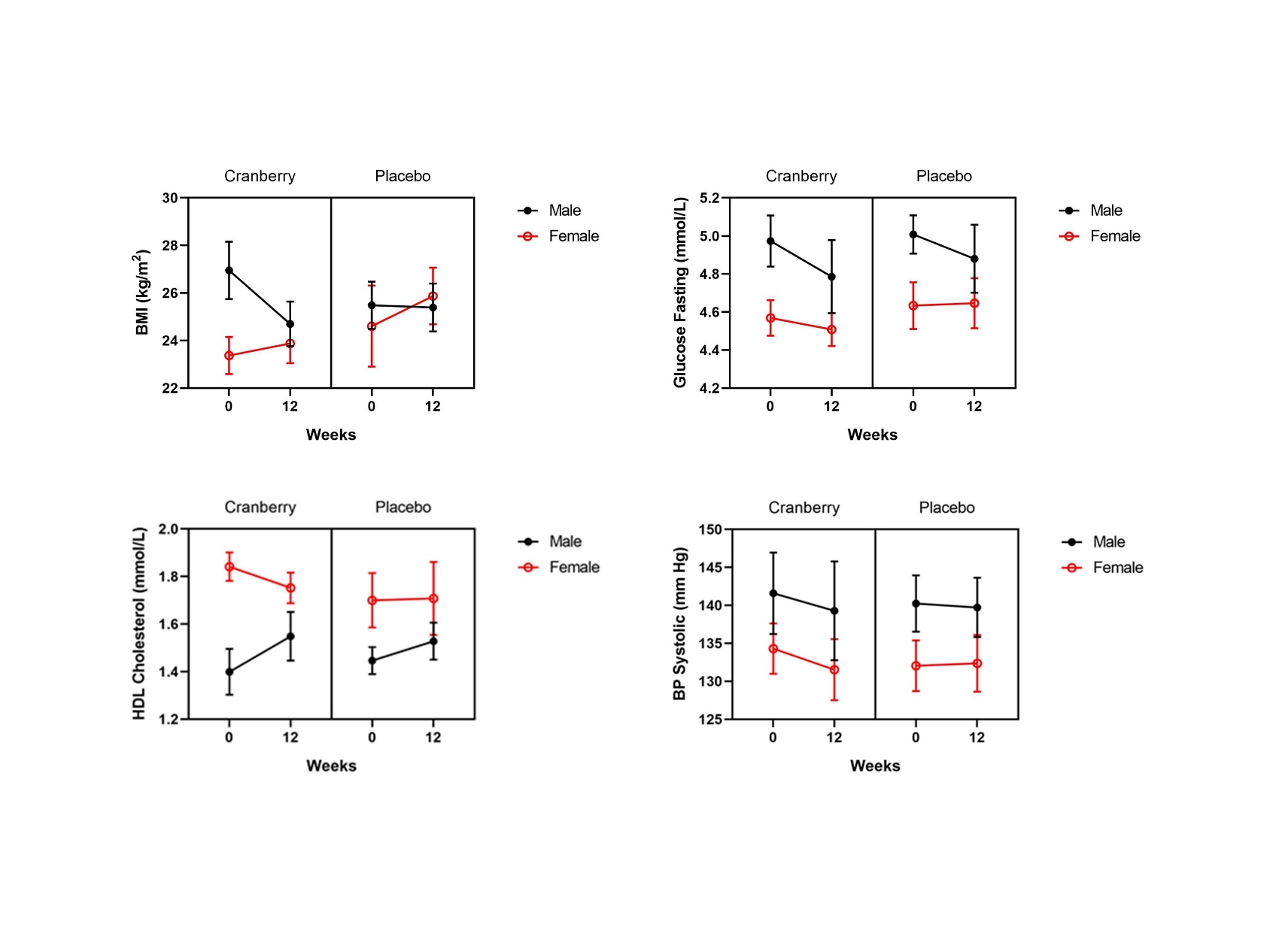
**

**Supplementary Figure S3**: Impact of cranberries and placebo on circulating concentrations of the brain derived neurotrophic factor (BDNF) over a 12-week period and as measured by ELISA in participants sera. No significant changes were observed.

**
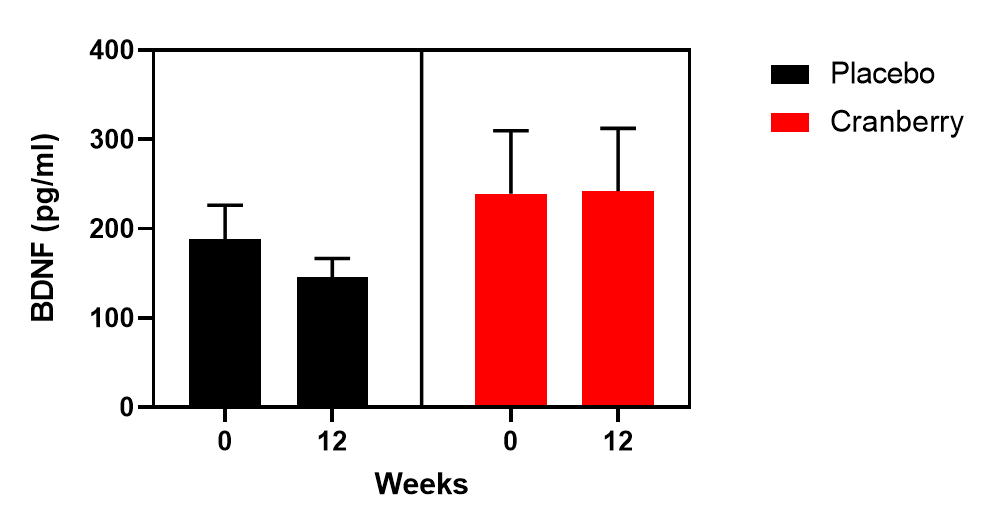
**

**Supplementary Table 1.** Product specifications for the freeze-dried cranberry powder.

| **Specifications** | **Characteristic or Amount** | **Method** | **Reference Standard** |
| --- | --- | --- | --- |
| Appearance | Deep red | Visual | Fresh cranberry |
| Flavour | Typical | Sensory | Fresh cranberry |
| % Moisture | 2% |  | Fresh cranberry |
| Screen Analysis | 100% through U.S. #20 screen | VDF FC 2 | 100% through U.S. #20 screen |
| Total Polyphenols | 17.48 mg/g | Folin-Ciocalteu | Gallic acid equivalents |
| Tartaric Acid | <200 ppm | HPLC | Tartaric Acid |
| Citric acid | 83,900 ppm | HPLC | Citric acid |
| Fumaric acid | <200 ppm | HPLC | Fumaric acid |
| Malic acid | 63,000 ppm | HPLC | Malic acid |
| Quinic acid | 86,600 ppm | HPLC | Quinic acid |
| Crude protein | 2.09% | Kjeldahl | Not applicable |
| Acid Det. fibre | 15.58% | NDF/ADF/Lignin | Not applicable |
| Neutral Detergent Fibre | 16.15% | NDF/ADF/Lignin | Not applicable |
| Calcium | 0.04% | ICP-AES | Calcium |
| Phosphorus | 0.06% | ICP-AES | Phosphorus |
| Magnesium | 0.05% | ICP-AES | Magnesium |
| Potassium | 0.69% | ICP-AES | Potassium |
| Sulphur | 0.05% | ICP-AES | Sulphur |
| Fat | 2.98% | Ether extraction | Not applicable |
| Ash | 4.10% | Combustion | Not applicable |
| Hydrophilic antioxidant activity | 214 umol TE/g | ORAC_S:8 | Trolox equivalents |
| In vitro anti-adhesion activity | 7.5 mg/mL | MRHA | Minimum Inhibitory Concentration |

**Supplementary Table 2**: Quantification of (poly)phenolic compounds in the cranberry powder*.* Polyphenols were quantified using uHPLC–MSn analysis as described in Supplementary methods.

| (9 grams, 2 sachets of 4.5 grams) | Quantity (mg) |
| --- | --- |
| **Sum of Polyphenols** | **587.5** |
| **Total Proanthocyanidins (PACs), mg** | **374.6** |
| Soluble PACs (c-PAC, DMAC)  Insoluble PACs (BuOH-HCl)  Epicatechin (LC-MS)  Catechin (LC-MS) | 280.8  93.4  0.387  0.072 |
| **Total Flavonols, mg** | **84.6** |
| Quercetin-3-rhamnoside eq., mg (HPLC)  Quercetin, mg (LC-MS)  Kaempferol, mg (LC-MS)  Isorhamnetin (LC-MS)  Myricetin (LC-MS)  Laricitrin (LC-MS)  Syringetin (LC-MS) | 81  2.3  0.045  0.34  0.64  0.08  0.23 |
| **Total Anthocyanins** | **58.5** |
| Cyanidin-3-*O*-galactoside (LC-MS)  Cyanidin-3-*O*-arabinoside (LC-MS)  Peonidin-3-*O*-galactoside (LC-MS)  Peonidin-3-*O*-arabinoside (LC-MS) | 6.8  3.8  35.0  12.9 |
| **Total Phenolic acids** | **69.8** |
| Benzoic acid (LC-MS)  3,4-Dihydroxybenzoic acid (Protocatechuic acid) (LC-MS)  Cinnamic acid (LC-MS)  p-Coumaric acid (LC-MS)  Caffeic acid (LC-MS)  5-Caffeoylquinic acid (LC-MS)  Coumaroyl-glucose (LC-MS)  Feruloyl-glucose (LC-MS)  Feruoylquinic acid(LC-MS)  Sinapoyl-glucose (LC-MS)  Caffeoyl-glucose I (LC-MS)  Caffeoyl-glucose II (LC-MS)  Glucosyl-caffeoyl-glucose (LC-MS) | 2.3  6.9  2.0  1.9  0.8  4.7  24.3  8.9  0.4  3.6  2.4  4.8  6.8 |

BuOH-HCl: butanol – hydrochloric acid; c-PAC: cranberry proanthocyanidins; DMAC: 4-(Dimethylamino)cinnamaldehyde; HPLC: High-performance liquid chromatography; LC-MS: Liquid chromatography–mass spectrometry; PAC: proanthocyanidins.

**Supplementary Table 3**: Background diet as established by the food frequency questionnaire (FFQ)

|  | **Placebo** | | **Cranberry** | |  |
| --- | --- | --- | --- | --- | --- |
|  | ***Mean*** | ***SD*** | ***Mean*** | ***SD*** | ***Sig. (p)*** |
| **Proteins (g/d)** | **78.1** | **25.4** | **84.7** | **30.0** | **0.38** |
| **Carbohydrates (g/d)** | **244.6** | **87.8** | **256.5** | **107.9** | **0.65** |
| Sugar (g/d) | 126.7 | 60.1 | 126.9 | 57.0 | 0.99 |
| Glucose (g/d) | 25.6 | 20.0 | 22.2 | 9.2 | 0.41 |
| Fructose (g/d) | 28.4 | 21.1 | 24.6 | 9.8 | 0.39 |
| Sucrose (g/d) | 45.5 | 17.4 | 46.9 | 22.0 | 0.79 |
| Fibres (g/d) | 21.9 | 11.2 | 21.4 | 9.1 | 0.84 |
| AOAC Fibres (g/d) | 6.9 | 4.4 | 5.7 | 3.6 | 0.27 |
| Starch (g/d) | 114.3 | 39.8 | 125.1 | 61.0 | 0.44 |
| **Fat (g/d)** | **78.6** | **29.8** | **85.9** | **33.5** | **0.39** |
| SFA (g/d) | 29.3 | 11.6 | 32.1 | 13.4 | 0.40 |
| MUFA (g/d) | 29.7 | 9.3 | 27.7 | 9.3 | 0.68 |
| PUFA (g/d) | 13.4 | 4.9 | 13.5 | 4.6 | 0.94 |
| TRANS (g/d) | 2.1 | 0.9 | 2.2 | 1.2 | 0.55 |
| **Energy** |  |  |  |  |  |
| kCal/d | 2007.0 | 645.3 | 2126.0 | 758.6 | 0.53 |
| kJ/d | 8467.0 | 2710.0 | 8963.0 | 3184.0 | 0.53 |
| **Vitamins** |  |  |  |  |  |
| Vitamin C (mg/d) | 131.7 | 67.0 | 116.4 | 50.2 | 0.34 |
| Vitamin D (ug/d) | 3.5 | 1.8 | 5.0 | 3.0 | **0.02** |
| Vitamin E (mg/d) | 11.0 | 4.2 | 12.4 | 5.9 | 0.31 |
| Vitamin B6 (mg/d) | 2.14 | 0.6 | 2.35 | 0.9 | 0.30 |
| Niacin (mg/d) | 18.9 | 5.7 | 19.9 | 7.3 | 0.56 |
| Folic acid (ug/d) | 359.8 | 127.2 | 353.0 | 153.2 | 0.86 |
| Vitamin B12 (ug/d) | 5.8 | 2.5 | 7.4 | 3.5 | 0.06 |
| ***Flavonoids and caffeine*** |  |  |  |  |  |
| Flavonols (mg/d) | 39.6 | 35.4 | 35.2 | 18.7 | 0.55 |
| Flavan-3-ols (mg/d) | 116 | 86.8 | 117.9 | 90.15 | 0.93 |
| Flavones (mg/d) | 3.9 | 12.7 | 2.2 | 2.9 | 0.49 |
| Procyanidins (mg/d) | 36.9 | 24.0 | 34.4 | 24.6 | 0.69 |
| Flavanones (mg/d) | 32.0 | 29.7 | 27 | 20.2 | 0.46 |
| Caffeine (mg/d) | 189.7 | 91.1 | 241.0 | 130.0 | 0.09 |
| ***Others*** |  |  |  |  |  |
| DQI | 41.0 | 11.3 | 40.0 | 9.8 | 0.71 |
| Alcohol (g/d) | 9.9 | 9.4 | 7.9 | 8.6 | 0.39 |

DQI: Diet Quality Index (component of the DQI include fruit and vegetable intake, oily fish, white fish, red and processed meat, total fat intake, saturated fat intake and fibres (Craig, McNeill, Masson, & Macdiarmid, 2016). The alcohol component is not included); MUFA: Mono-unsaturated fatty acid; PUFA: Poly-unsaturated fatty acid; SFA: Saturated fatty acids.

**Supplementary Table 4**. Mean regional perfusion at baseline and follow-up for cranberry and placebo groups, and significance of baseline and within group differences between baseline and follow-up for each region, and group x time interaction.

|  |  |  | Baseline perfusion (mL/min/100g) | | Baseline differences | Follow-up perfusion (mL/min/100g) | | Group x time |
| --- | --- | --- | --- | --- | --- | --- | --- | --- |
| Region |  | Group | M | SD | Sig. | M | SD | Sig. |
| Thalamus | Left | Cranberry | 51.578 | 9.598 | .259 | 53.910 | 11.123 | .129 |
|  |  | Placebo | 44.431 | 8.918 |  | 42.314 | 8.823 |  |
|  | Right | Cranberry | 51.429 | 8.949 | .260 | 52.374 | 11.111 | .317 |
|  |  | Placebo | 45.563 | 8.695 |  | 43.529 | 7.828 |  |
| Caudate | Left | Cranberry | 41.625 | 5.822 | .925 | 44.318 | 6.918 | .132 |
|  |  | Placebo | 40.566 | 7.649 |  | 39.641 | 5.954 |  |
|  | Right | Cranberry | 42.509 | 6.252 | .960 | 44.972 | 7.868 | **.049** |
|  |  | Placebo | 41.113 | 8.981 |  | 38.824 | 5.871 |  |
| Putamen | Left | Cranberry | 46.107 | 7.083 | .896 | 47.883 | 6.765 | .181 |
|  |  | Placebo | 44.737 | 7.647 |  | 43.018 | 4.808 |  |
|  | Right | Cranberry | 44.812 | 6.426 | .674 | 46.849 | 8.228 | .149 |
|  |  | Placebo | 43.138 | 5.914 |  | 40.787 | 4.672 |  |
| Pallidum | Left | Cranberry | 35.305 | 5.301 | .356 | 36.777 | 5.391 | .393 |
|  |  | Placebo | 33.046 | 4.472 |  | 33.025 | 3.248 |  |
|  | Right | Cranberry | 37.185 | 5.668 | .261 | 38.213 | 6.663 | .096 |
|  |  | Placebo | 34.163 | 5.073 |  | 31.734 | 4.044 |  |
| Brain Stem |  | Cranberry | 40.585 | 8.405 | .306 | 41.429 | 8.753 | .292 |
|  |  | Placebo | 36.219 | 5.600 |  | 34.643 | 6.047 |  |
| Hippocampus | Left | Cranberry | 50.911 | 8.701 | .234 | 53.381 | 10.996 | .313 |
|  |  | Placebo | 44.128 | 6.016 |  | 43.989 | 7.106 |  |
|  | Right | Cranberry | 51.699 | 9.006 | .354 | 52.977 | 10.891 | .249 |
|  |  | Placebo | 45.607 | 6.451 |  | 43.878 | 5.710 |  |
| Amygdala | Left | Cranberry | 47.780 | 7.803 | .506 | 49.688 | 9.087 | .237 |
|  |  | Placebo | 43.417 | 5.377 |  | 41.844 | 6.023 |  |
|  | Right | Cranberry | 46.878 | 8.542 | .638 | 49.685 | 10.671 | .088 |
|  |  | Placebo | 43.494 | 6.802 |  | 41.429 | 5.831 |  |
| Accumbens Area | Left | Cranberry | 44.759 | 7.669 | .637 | 46.807 | 7.658 | .133 |
|  |  | Placebo | 42.644 | 8.098 |  | 40.543 | 7.401 |  |
|  | Right | Cranberry | 44.308 | 7.319 | .929 | 48.015 | 9.337 | **.034** |
|  |  | Placebo | 44.383 | 10.095 |  | 41.014 | 7.017 |  |
| Ventral Diancephalon | Left | Cranberry | 42.059 | 6.513 | .192 | 43.763 | 8.304 | .057 |
|  |  | Placebo | 37.505 | 5.119 |  | 35.154 | 5.521 |  |
|  | Right | Cranberry | 42.796 | 6.213 | .259 | 43.460 | 8.202 | .171 |
|  |  | Placebo | 38.509 | 6.531 |  | 35.994 | 5.202 |  |
| Choroid Plexus | Left | Cranberry | 50.926 | 14.603 | .510 | 52.926 | 18.117 | .321 |
|  |  | Placebo | 41.436 | 11.535 |  | 40.234 | 7.995 |  |
|  | Right | Cranberry | 50.742 | 11.528 | .374 | 53.262 | 15.572 | .207 |
|  |  | Placebo | 42.066 | 8.667 |  | 40.938 | 7.198 |  |
| Caudal anterior cingulate | Left | Cranberry | 53.247 | 7.600 | .990 | 56.506 | 9.544 | .207 |
|  |  | Placebo | 50.731 | 10.893 |  | 50.751 | 8.957 |  |
|  | Right | Cranberry | 53.930 | 7.917 | .940 | 56.538 | 11.119 | .224 |
|  |  | Placebo | 51.802 | 11.037 |  | 51.337 | 9.003 |  |
| Caudal middle frontal | Left | Cranberry | 50.833 | 8.958 | .747 | 52.708 | 10.544 | .406 |
|  |  | Placebo | 48.799 | 11.437 |  | 48.882 | 10.233 |  |
|  | Right | Cranberry | 52.407 | 9.518 | .715 | 55.648 | 13.623 | .213 |
|  |  | Placebo | 47.331 | 11.583 |  | 46.658 | 10.792 |  |
| Cuneus | Left | Cranberry | 52.986 | 12.434 | .390 | 55.851 | 14.406 | .331 |
|  |  | Placebo | 44.465 | 11.071 |  | 44.304 | 13.331 |  |
|  | Right | Cranberry | 55.969 | 13.078 | .424 | 59.485 | 16.229 | .332 |
|  |  | Placebo | 49.640 | 10.730 |  | 49.275 | 9.835 |  |
| Entorhinal cortex | Left | Cranberry | 43.825 | 7.062 | .425 | 46.333 | 8.547 | .162 |
|  |  | Placebo | 38.689 | 4.819 |  | 38.240 | 5.911 |  |
|  | Right | Cranberry | 43.115 | 8.401 | .461 | 46.675 | 9.917 | **.030** |
|  |  | Placebo | 40.149 | 6.024 |  | 37.734 | 5.564 |  |
| fusiform | Left | Cranberry | 42.003 | 7.871 | .494 | 44.464 | 9.811 | .122 |
|  |  | Placebo | 37.679 | 7.384 |  | 36.268 | 9.624 |  |
|  | Right | Cranberry | 42.817 | 8.650 | .466 | 44.928 | 10.072 | .271 |
|  |  | Placebo | 39.117 | 6.503 |  | 38.386 | 5.024 |  |
| Inferior parietal | Left | Cranberry | 47.184 | 7.824 | .701 | 49.956 | 11.035 | .265 |
|  |  | Placebo | 43.726 | 8.267 |  | 43.177 | 11.028 |  |
|  | Right | Cranberry | 59.820 | 10.177 | .962 | 63.779 | 15.628 | .154 |
|  |  | Placebo | 55.973 | 11.981 |  | 53.647 | 11.060 |  |
| Inferior temporal | Left | Cranberry | 38.540 | 6.192 | .616 | 40.294 | 7.726 | .222 |
|  |  | Placebo | 35.520 | 7.550 |  | 34.697 | 7.985 |  |
|  | Right | Cranberry | 41.638 | 7.774 | .844 | 44.406 | 9.814 | .115 |
|  |  | Placebo | 39.635 | 7.608 |  | 38.100 | 6.961 |  |
| Isthmus cingulate | Left | Cranberry | 66.546 | 13.345 | .695 | 69.520 | 15.069 | .302 |
|  |  | Placebo | 61.114 | 10.806 |  | 59.208 | 12.476 |  |
|  | Right | Cranberry | 68.620 | 13.761 | .845 | 71.803 | 16.386 | .253 |
|  |  | Placebo | 64.506 | 12.110 |  | 62.240 | 10.983 |  |
| Lateral occipital | Left | Cranberry | 39.202 | 9.371 | .879 | 42.773 | 11.749 | .129 |
|  |  | Placebo | 35.625 | 8.270 |  | 34.744 | 9.939 |  |
|  | Right | Cranberry | 45.882 | 11.182 | .921 | 50.562 | 15.859 | .123 |
|  |  | Placebo | 41.984 | 9.160 |  | 40.652 | 8.124 |  |
| Lateral orbitofrontal | Left | Cranberry | 43.114 | 7.661 | .837 | 44.847 | 7.216 | .188 |
|  |  | Placebo | 40.917 | 5.183 |  | 39.854 | 5.459 |  |
|  | Right | Cranberry | 42.739 | 7.765 | .890 | 45.286 | 8.576 | .089 |
|  |  | Placebo | 41.205 | 5.714 |  | 39.276 | 6.397 |  |
| lingual | Left | Cranberry | 49.388 | 10.867 | .318 | 51.645 | 12.398 | .266 |
|  |  | Placebo | 41.421 | 7.520 |  | 40.694 | 10.058 |  |
|  | Right | Cranberry | 50.806 | 10.420 | .576 | 53.480 | 12.907 | .225 |
|  |  | Placebo | 45.920 | 9.364 |  | 44.870 | 7.006 |  |
| Medial orbitofrontal | Left | Cranberry | 41.078 | 7.943 | .654 | 43.394 | 7.981 | .115 |
|  |  | Placebo | 38.210 | 4.260 |  | 37.010 | 4.826 |  |
|  | Right | Cranberry | 40.704 | 7.305 | .799 | 42.796 | 7.850 | .065 |
|  |  | Placebo | 38.726 | 5.236 |  | 36.426 | 4.148 |  |
| Middle temporal | Left | Cranberry | 46.394 | 6.380 | .950 | 48.196 | 8.513 | .266 |
|  |  | Placebo | 44.526 | 8.315 |  | 43.453 | 8.535 |  |
|  | Right | Cranberry | 53.777 | 8.655 | .838 | 56.461 | 11.884 | .103 |
|  |  | Placebo | 52.072 | 8.859 |  | 48.940 | 8.483 |  |
| parahippocampal | Left | Cranberry | 44.595 | 7.659 | .245 | 46.262 | 9.611 | .170 |
|  |  | Placebo | 38.904 | 5.212 |  | 37.329 | 7.696 |  |
|  | Right | Cranberry | 44.581 | 7.982 | .411 | 45.350 | 8.678 | .310 |
|  |  | Placebo | 39.701 | 5.334 |  | 38.526 | 4.421 |  |
| paracentral | Left | Cranberry | 61.648 | 10.858 | .313 | 64.278 | 12.569 | .378 |
|  |  | Placebo | 54.794 | 9.812 |  | 55.410 | 7.592 |  |
|  | Right | Cranberry | 63.752 | 11.427 | .383 | 67.322 | 14.730 | .264 |
|  |  | Placebo | 56.897 | 10.851 |  | 56.665 | 8.306 |  |
| Pars opercularis | Left | Cranberry | 55.308 | 8.351 | .684 | 56.906 | 9.674 | .229 |
|  |  | Placebo | 54.346 | 9.215 |  | 52.959 | 8.009 |  |
|  | Right | Cranberry | 57.554 | 8.166 | .956 | 59.771 | 10.623 | .168 |
|  |  | Placebo | 55.450 | 9.317 |  | 53.019 | 7.739 |  |
| Pars orbitalis | Left | Cranberry | 48.249 | 7.945 | .368 | 49.454 | 8.426 | .147 |
|  |  | Placebo | 48.166 | 5.689 |  | 45.967 | 7.621 |  |
|  | Right | Cranberry | 50.413 | 7.010 | .913 | 52.484 | 9.601 | .134 |
|  |  | Placebo | 49.480 | 6.763 |  | 46.806 | 7.974 |  |
| Pars triangularis | Left | Cranberry | 51.841 | 8.423 | .665 | 53.179 | 8.389 | .294 |
|  |  | Placebo | 50.467 | 7.694 |  | 49.272 | 7.846 |  |
|  | Right | Cranberry | 52.363 | 52.363 | .703 | 54.465 | 9.283 | .225 |
|  |  | Placebo | 52.437 | 8.628 |  | 50.395 | 7.429 |  |
| pericalcarine | Left | Cranberry | 54.206 | 13.153 | .311 | 55.924 | 15.300 | .422 |
|  |  | Placebo | 44.076 | 11.313 |  | 43.734 | 12.717 |  |
|  | Right | Cranberry | 56.682 | 13.398 | .390 | 59.092 | 17.183 | .511 |
|  |  | Placebo | 49.551 | 11.586 |  | 50.065 | 10.309 |  |
| postcentral | Left | Cranberry | 47.319 | 7.669 | .835 | 49.260 | 9.147 | .404 |
|  |  | Placebo | 45.345 | 7.656 |  | 45.503 | 8.657 |  |
|  | Right | Cranberry | 53.635 | 10.032 | .846 | 56.484 | 13.291 | .239 |
|  |  | Placebo | 50.000 | 9.127 |  | 49.122 | 8.050 |  |
| Posterior cingulate | Left | Cranberry | 69.584 | 10.615 | .471 | 73.005 | 13.098 | .305 |
|  |  | Placebo | 62.915 | 13.411 |  | 62.978 | 11.728 |  |
|  | Right | Cranberry | 71.630 | 10.091 | .557 | 75.673 | 13.394 | .179 |
|  |  | Placebo | 65.401 | 13.447 |  | 64.275 | 11.540 |  |
| precentral | Left | Cranberry | 50.074 | 8.062 | .841 | 52.159 | 9.343 | .322 |
|  |  | Placebo | 47.759 | 8.797 |  | 47.856 | 8.982 |  |
|  | Right | Cranberry | 54.395 | 9.558 | .642 | 57.293 | 12.748 | .190 |
|  |  | Placebo | 49.980 | 9.244 |  | 48.946 | 8.589 |  |
| precuneus | Left | Cranberry | 65.048 | 12.161 | .394 | 68.248 | 15.603 | .303 |
|  |  | Placebo | 57.527 | 11.394 |  | 56.561 | 12.953 |  |
|  | Right | Cranberry | 66.277 | 13.042 | .574 | 69.769 | 17.425 | .359 |
|  |  | Placebo | 59.796 | 12.338 |  | 59.729 | 12.065 |  |
| Rostral anterior cingulate | Left | Cranberry | 52.766 | 10.183 | .910 | 55.630 | 10.427 | .192 |
|  |  | Placebo | 50.362 | 9.350 |  | 49.547 | 8.104 |  |
|  | Right | Cranberry | 51.239 | 9.402 | .677 | 53.613 | 10.447 | .154 |
|  |  | Placebo | 50.171 | 9.005 |  | 48.307 | 8.241 |  |
| Rostral middle frontal | Left | Cranberry | 51.581 | 9.785 | .491 | 53.173 | 10.436 | .306 |
|  |  | Placebo | 51.271 | 10.971 |  | 49.935 | 10.924 |  |
|  | Right | Cranberry | 51.296 | 9.418 | .865 | 53.413 | 13.013 | .234 |
|  |  | Placebo | 49.402 | 10.022 |  | 47.830 | 10.093 |  |
| Superior frontal | Left | Cranberry | 49.854 | 9.516 | .860 | 51.644 | 10.106 | .328 |
|  |  | Placebo | 47.229 | 9.531 |  | 46.770 | 8.719 |  |
|  | Right | Cranberry | 50.401 | 9.438 | .964 | 52.421 | 11.388 | .194 |
|  |  | Placebo | 47.418 | 9.950 |  | 46.018 | 8.638 |  |
| Superior parietal | Left | Cranberry | 43.022 | 8.966 | .703 | 46.786 | 12.981 | .224 |
|  |  | Placebo | 38.798 | 8.122 |  | 39.028 | 10.275 |  |
|  | Right | Cranberry | 49.990 | 11.498 | .612 | 54.170 | 16.816 | .226 |
|  |  | Placebo | 45.014 | 11.217 |  | 44.597 | 11.239 |  |
| Superior temporal | Left | Cranberry | 46.966 | 6.273 | .744 | 49.081 | 7.259 | .238 |
|  |  | Placebo | 45.938 | 6.156 |  | 45.183 | 7.381 |  |
|  | Right | Cranberry | 52.328 | 7.999 | .719 | 55.103 | 10.033 | .082 |
|  |  | Placebo | 51.418 | 7.240 |  | 48.596 | 7.194 |  |
| supramarginal | Left | Cranberry | 49.229 | 6.560 | .760 | 51.203 | 8.007 | .325 |
|  |  | Placebo | 48.050 | 8.427 |  | 47.551 | 9.250 |  |
|  | Right | Cranberry | 59.367 | 9.102 | .881 | 62.928 | 12.679 | .104 |
|  |  | Placebo | 56.949 | 11.830 |  | 54.386 | 9.487 |  |
| Transverse temporal | Left | Cranberry | 61.465 | 8.057 | .729 | 65.014 | 9.404 | .231 |
|  |  | Placebo | 60.435 | 8.226 |  | 60.360 | 9.284 |  |
|  | Right | Cranberry | 65.395 | 10.405 | .940 | 67.556 | 11.845 | .119 |
|  |  | Placebo | 62.050 | 9.454 |  | 58.439 | 8.475 |  |
| insula | Left | Cranberry | 52.786 | 7.791 | .779 | 56.297 | 9.309 | .163 |
|  |  | Placebo | 52.393 | 8.502 |  | 52.064 | 8.125 |  |
|  | Right | Cranberry | 55.707 | 8.726 | .945 | 58.964 | 11.772 | .056 |
|  |  | Placebo | 53.947 | 7.570 |  | 50.853 | 7.000 |  |

**References**

Craig, L. C. A., McNeill, G., Masson, L. F., & Macdiarmid, J. I. (2016). Diet Quality Index in children in Scotland: associations with age, sex, socio-economic deprivation and obesity. *Proceedings of the Nutrition Society, 75*(OCE3). doi:10.1017/s0029665116002007

Mena, P., Sánchez-Salcedo, E. M., Tassotti, M., Martínez, J. J., Hernández, F., & Del Rio, D. (2016). Phytochemical evaluation of eight white (Morus alba L.) and black (Morus nigra L.) mulberry clones grown in Spain based on UHPLC-ESI-MSn metabolomic profiles. *Food research international, 89*, 1116-1122.
